# Pulmonary inflammation and fibroblast immunoregulation: from bench to bedside

**DOI:** 10.1172/JCI170499

**Published:** 2023-09-01

**Authors:** Mohamed A. Ghonim, David F. Boyd, Tim Flerlage, Paul G. Thomas

**Affiliations:** 1 Department of Immunology, St. Jude Children’s Research Hospital, Memphis, Tennessee, USA.; 2 Department of Microbiology and Immunology, Faculty of Pharmacy, Al Azhar University, Cairo, Egypt.; 3 Molecular, Cell and Developmental Biology, University of California, Santa Cruz, Santa Cruz, California, USA.; 4 Department of Infectious Diseases, St. Jude Children’s Research Hospital, Memphis, Tennessee, USA.

## Abstract

In recent years, there has been an explosion of interest in how fibroblasts initiate, sustain, and resolve inflammation across disease states. Fibroblasts contain heterogeneous subsets with diverse functionality. The phenotypes of these populations vary depending on their spatial distribution within the tissue and the immunopathologic cues contributing to disease progression. In addition to their roles in structurally supporting organs and remodeling tissue, fibroblasts mediate critical interactions with diverse immune cells. These interactions have important implications for defining mechanisms of disease and identifying potential therapeutic targets. Fibroblasts in the respiratory tract, in particular, determine the severity and outcome of numerous acute and chronic lung diseases, including asthma, chronic obstructive pulmonary disease, acute respiratory distress syndrome, and idiopathic pulmonary fibrosis. Here, we review recent studies defining the spatiotemporal identity of the lung-derived fibroblasts and the mechanisms by which these subsets regulate immune responses to insult exposures and highlight past, current, and future therapeutic targets with relevance to fibroblast biology in the context of acute and chronic human respiratory diseases. This perspective highlights the importance of tissue context in defining fibroblast-immune crosstalk and paves the way for identifying therapeutic approaches to benefit patients with acute and chronic pulmonary disorders.

## Frameworks for defining lung fibroblast heterogeneity

### Phenotypic heterogeneity of lung fibroblasts.

Fibroblasts in the respiratory tract represent a diverse group of cells with a high degree of plasticity. Their plasticity presents several challenges in studying specific functions, including homeostatic, immune-regulatory, tissue-repair, and fibrotic functions, which vary in different respiratory diseases. The lack of consistent nomenclature and of consensus genetic and/or protein markers has further complicated interpretation of findings and attribution of cellular activities to specific cell types. Fibroblasts belong to a broader group of related cells, referred to as “mesenchymal” and in some cases “stromal.” Mesenchymal cells are frequently identified by lack of lineage markers defining other major cell types in the respiratory tract, including epithelial cells (EpCAM, also called CD326), endothelial cells (PECAM1, also called CD31), and immune cells (PTPRC, also called CD45). In the respiratory tract, these mesenchymal cells include fibroblasts, bronchial smooth muscle cells, vascular smooth muscle cells, and pericytes. The expression of extracellular matrix–related (ECM-related) genes, including those encoding structural proteins (collagens, proteoglycans, and other glycoproteins) and ECM-modifying proteases, characterizes mesenchymal cells. However, the specific genes expressed vary across cell types and depend on experimental conditions, treatments, and disease context.

Epithelial cells also undergo epithelial-mesenchymal transition (EMT), a process in which epithelial characteristics are downregulated and fibroblast phenotypes are acquired. There is a wide range of EMT phenotypic presentations, where changes in gene expression and posttranslational regulatory mechanisms take place to enable induction of fibroblast-like cytoarchitecture and migratory capability (1). Several studies reported a role for EMT in the etiology of lung disorders, through mediating developmental abnormalities, tissue fibrosis, and remodeling, suggesting a correlation between EMT and progression of pulmonary diseases, including asthma, chronic obstructive pulmonary disease (COPD), and lung fibrosis (2–4).

Recent comparative studies across tissues identified common transcriptional programs associated with the term “universal” fibroblast types that show shared inflammatory activation states across diseases (5, 6). In addition to these common fibroblast identities across tissues, there are also tissue-specific transcriptional programs consistent with the roles for these cells in generating distinct tissue structures in compartmentalized organs such as the lungs. Impressively, both universal and tissue-specific identities are conserved across mice and humans. Several different frameworks have been developed to classify fibroblasts and compare their functions in lung development, injury, and repair.

### Functional heterogeneity of lung fibroblasts.

One established framework classifies fibroblasts on the basis of function during homeostasis, denoting fibroblasts with contractile functions as myofibroblasts, those with storage functions as lipofibroblasts, and those with synthesis functions as matrix fibroblasts (7). Myofibroblasts exhibit a contractile phenotype associated with activation by a number of different stimuli, including TGF-β. Myofibroblasts have well-studied functions in tissue repair and can adopt pathologic functions during fibrosis development (8). Lipofibroblasts are mesenchymal cells located in close proximity to type 2 alveolar airway epithelial cells (T2 AECs) that contain lipid vesicles and express adipose-related genes (9). Lipofibroblasts play functional roles in supporting T2 AECs during pulmonary development and injury via production of surfactants and retinoic acids (10, 11). Matrix fibroblasts inhabit the lung interstitial ECM and play roles in generating and modifying lung ECM. These cells can additionally adopt activation states depending on microenvironmental cues in the tissue. Consistent with the high degree of plasticity among fibroblasts, there is evidence that fibroblasts can convert between lipofibroblast and myofibroblast functional types, which impact resolution of lung injury and repair (12).

There is further heterogeneity among mesenchymal cells in the respiratory tract with distinct cell types occupying restricted anatomical niches. Tsukui et al. identified peribronchial fibroblasts, another fibroblast subtype, transcriptionally related to, but distinct from, smooth muscle cells and located in subepithelial lining of mouse bronchioles (13). Fibroblasts with transcriptional profiles similar to those of peribronchial fibroblasts, marked by high expression of Hedgehog-interacting protein (HHIP) (14), have been identified by other groups, but were classified as myofibroblasts (15, 16). It is unclear whether there is a human homolog of the HHIP-expressing peribronchial fibroblasts. At least one study has identified a distinct population of mesenchymal cells with similar peribronchial, subepithelial localization in human lungs (17). This population shares similar transcriptional profiles to the peribronchial fibroblasts in mice, including expression of *LGR5* and *ASPN*. A notable difference, however, is lack of *HHIP* expression. Considering the evidence that HHIP plays an important functional role in peribronchial fibroblasts modulating tissue inflammation associated with COPD (16), it is unclear whether LGR5^+^ fibroblasts in humans and HHIP^+^ fibroblasts in mice have homologous functions.

### Spatial heterogeneity of lung fibroblasts.

Another framework developed to classify fibroblasts is based on anatomical location in the respiratory tract. This framework organizes distinct fibroblast types according to the compartment in which they reside, in a proximal, termed “adventitial,” to distal, termed “alveolar,” orientation. Organizing fibroblast types with respect to anatomical location is particularly useful for (a) identifying distinct activation states among various subtypes, which differentially regulate response to injury/insult, and (b) identifying precursor cells that can adopt these states and/or fates. Single-cell gene expression studies in both mice and humans have consistently identified at least two different broad types of *Pdgfra*-expressing fibroblasts, representing proximal and distal transcriptional profiles, regardless of disease state. Imaging of lung tissue with RNA in situ hybridization or antibodies recognizing protein markers demonstrates that these distinct fibroblast subtypes are localized either to proximal areas near conducting airways, termed “bronchi,” and blood vessels, or to the distal alveolar regions. A number of groups have adopted the nomenclature “proximal” (also called “adventitial”) versus “alveolar” to characterize these distinct subtypes (13, 17–26) (Table 1).

Narvaez Del Pilar et al. recently built on this proximal-distal framework by adding another layer of specificity based on cell types with which mesenchymal cells are most closely associated (15). Proximal fibroblasts exhibit enriched gene expression of cytokines and chemokines known to be important for organizing and activating immune cells (13). These gene expression profiles along with their localization in adventitial cuff regions suggest important roles in orchestrating immune cell trafficking and function (27). Distal fibroblasts share common transcriptional features with the lipofibroblast (13, 15) and express genes encoding BMP and FGFs, important for maintenance of alveolar epithelial cells (28).

Consistent with their distinct localization, proximal and alveolar fibroblasts differentially express genes encoding structural ECM proteins, including collagens, with *Col14a1* denoting proximal fibroblasts and *Col13a1* denoting alveolar fibroblasts (21, 29, 30). Despite differences in nomenclature and selection of lineage markers, results across single-cell gene expression studies have been remarkably consistent in distinguishing these two spatially restricted subsets.

Despite important differences in respiratory tract anatomy between mice and humans (31), comparative studies of lung fibroblasts based on single-cell gene expression demonstrate a surprising amount of conservation with respect to the proximal and adventitial framework and lineage markers — for example, expression of the mouse *Pi16* or the human *PI16* gene indicates adventitial fibroblasts, and expression of the mouse *Npnt* or the human *NPNT* gene indicates alveolar fibroblasts (5, 13, 20). Mesenchymal cells also undergo dramatic changes with respect to phenotype and function as they guide the development of the respiratory tract early in life (7, 15, 32–34). One salient example of mesenchymal cell dynamics is the transient presence of secondary crest myofibroblasts (SCMFs) during lung alveologenesis. SCMFs appear along septal ridges and help partition nascent alveoli, increasing surface area for gas exchange (31).

Changes in the lung mesenchymal compartment during development, and later in aging (35), add another layer of complexity for defining distinct lineages of cells with specific functions in lung development, injury, and repair. Mesenchymal plasticity and dynamics of defined lineages are important to consider when investigating age-dependent diseases — for example, severe respiratory viral disease, asthma, and pulmonary fibrosis — and assigning protective or pathologic functions to certain subtypes or activation states. Here, we will use the proximal-distal framework and refer to fibroblasts as “adventitial” and “alveolar” when reviewing the roles in lung inflammation and respiratory disease. It is becoming evident that lung fibroblasts can adopt a wide variety of activation states and assume diverse roles in regulating immune responses to insults. In addition to their well-established functions in tissue repair and fibrotic diseases, fibroblasts activate inflammatory pathways traditionally assigned to professional immune cells (5, 36). Lung fibroblasts can acquire inflammatory and/or pathologic functions in diverse respiratory disorders, including acute infections, allergy-related asthma, COPD, and fibrosis.

Fibroblasts have the capacity to respond to diverse signals and subsequently acquire different activation states. These signals include cell-intrinsic signals generated during infection or cellular perturbations and cell-extrinsic signals, including cytokines or growth factors. In addition to the extracellular cytokine environments that fibroblasts respond to, a specific fibroblast lineage — adventitial versus alveolar — may also determine their activation potential, in terms of magnitude and quality.

### Fibroblasts in primary and secondary lymphoid organs.

The relevance of crosstalk and interactions between fibroblasts and immune cells has clearly been defined by their function in both primary and secondary lymphoid organs.

In primary lymphoid organs, such as bone marrow, fibroblasts regulate hematopoietic stem and progenitor cell (HSPC) differentiation and maturation via expression of ECM proteins, which subsequently interact with integrins and CD44 on HSPC surfaces (37, 38). In this context, fibroblasts restrain the release of immature cells from the primary lymphoid structure into the circulation until full differentiation and maturation are achieved. This process is typically mediated by CXCL12 and VCAM-1 (37, 39).

**Figure 1:**
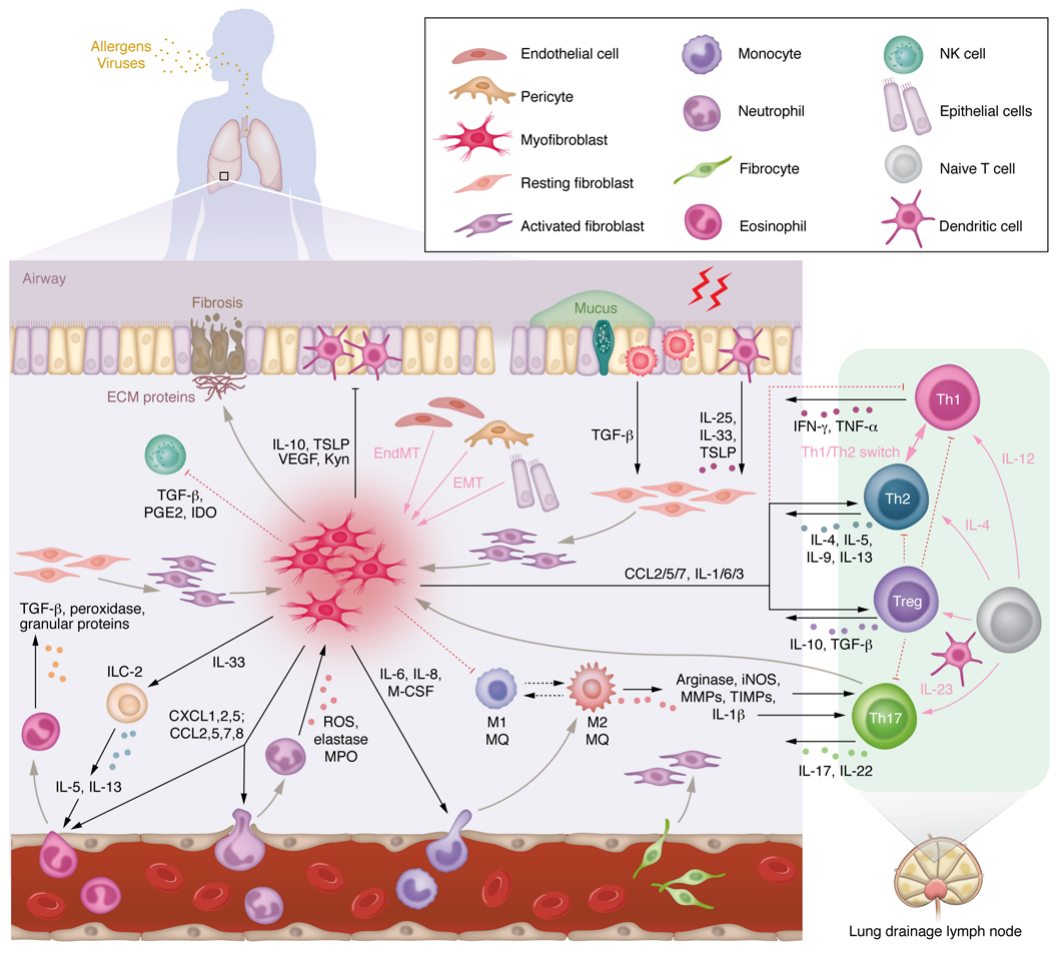
Lymphatic tissues in the lung regulate the differentiation and release of circulating leukocytes, while fibroblasts coordinate the convergence and communication between innate and adaptive immune cells. Lung insult caused by airway exposure to environmental triggers (e.g., aeroallergens, viral infection) induces a localized inflammatory response that drives the transformation of epithelial and endothelial cells to myofibroblasts through EMT and endothelial-mesenchymal transition (EndMT) processes. These processes also associate with the recruitment of circulating fibrocytes to lung ECM. Lung ECM-resident fibroblasts are simultaneously induced by a range of cytokines and growth factors, including IL-25, IL-33, TSLP, and TGF-β, and they transition from a resting state to an activated phenotype and finally to hypersecretory myofibroblasts. Myofibroblasts produce a range of cytokines and chemokines as well as soluble inflammatory factors, including IL-1, IL-6, IL-8, IL-13, M-CSF, CXCLs, CCLs, and TGF-β. These mediators regulate the infiltration, trafficking, and polarization of various adoptive and innate immune cells, including eosinophils, neutrophils, macrophages, and NK cells, as well as a variety of subsets of T lymphocytes, including Th1, Th2, and Th17 cells and Tregs. The subsequent blended matrix facilitates crosstalk and interaction between various immune subsets in the stromal tissue, further exacerbating the inflammatory cascade and promoting a tissue remodeling process.

Secondary lymphoid organs, such as lymph nodes, which are supported by highly specialized fibroblastic reticular cells (FRCs), are equally central to the development and activation of adaptive immunity (40). As compared with inflammatory fibroblasts, FRCs exhibit a transcriptional profile that is more enriched in genes from immune pathways contributing to antigen presentation and cytokine responses (36, 41). Within the lymphatic tissue, fibroblasts coordinate the direct interaction between innate and adaptive immune cells (Figure 1).

Myofibroblasts arising from mesenchymal tissue may also transition into specialized FRCs in lymph nodes, distinguishable from other immune subsets by the expression of podoplanin (PDPN) and PDGFRA and the absence of CD45 and CD31 expression (36, 42, 43). FRCs also express molecules shared by different types of inflammatory myofibroblasts, including desmin, vimentin, CD90, CD73, CD103, α-SMA, and ERTR7 (44). The stromal address code paradigm, known to guide leukocytes, includes localized fibroblasts and clearly reflects crosstalk through a network of soluble factors and adhesion molecules (45). Additional studies have also shown that FRCs could directly serve as antigen presenters to T and B cells, activating adaptive immunity and regulating self-reactive lymphocytes (46, 47).

Furthermore, FRCs have also been reported to provide a suppressive environment via different mechanisms, including antigenic tolerance and activating Tregs (48). Interestingly, single-cell gene expression analyses demonstrated several phenotypic distinctions among FRCs (49–51). Such structural and phenotypic heterogeneity among FRC subsets indicates contributions to antigen presentation, immune regulation, and tolerance.

## Immunoregulatory functions of fibroblasts in acute settings

While immunoregulatory functions of fibroblasts have been defined in primary and secondary lymphoid organs, much less is known in peripheral tissues. The presence of activated and inactivated subsets of fibroblasts has been linked to inflammatory responses through an influence on the proliferation, migration, residence, and apoptosis of infiltrating immune cells (52). The immunologic contribution of fibroblasts varies from recruitment and activation of immune cells to immunosuppression and clearance of inflammation, suggesting spatial heterogeneity and pivotal roles in orchestrating lung immune responses. Through production of surface molecules and leukocyte recruitment factors, fibroblasts signal their anatomical location to circulating immune cells, uniquely dictating the identity of recruited populations through various signaling mechanisms (45).

### Fibroblasts in acute inflammation and immune surveillance.

In addition to their critical role in coordinating immune functions within primary and secondary lymphoid organs, tissue-resident fibroblasts also exhibit an array of pathophysiologic functions in response to acute inflammation and infection. This coordination includes activation of COX-2, NF-κB–mediated kinases, and inflammasomes (53, 54). While cytokines and chemokines produced by lung-resident innate cells are crucial for initiating and maintaining inflammatory signaling and subsequent recruitment of T cells, lung fibroblasts have equally been shown to orchestrate the progression and resolution of inflammatory cascades in acute pulmonary inflammation via multiple mechanisms (55–57).

Upon exposure to inflammatory triggers, fibroblasts produce chemokines that promote immune cell recruitment. For example, expression of the CCR2 ligands CCL2 and CCL7 by fibroblasts facilitates myeloid infiltration into lungs (58). Once immune cells become locally confined in the ECM, fibroblasts additionally upregulate several proinflammatory mediators, including type I interferons, IL-6, B cell–activating factor (BAFF), and a proliferation-inducing ligand (APRIL), which convey survival signals to neighboring immune cells, allowing for further proliferation and activation (59–61). Furthermore, fibroblasts secrete multiple cytokines in the stromal compartment and serve as antigen-presenting cells activating both innate and adaptive machinery to support expansion, maintenance, and metabolic fitness of infiltrating memory T cells (50, 51, 62).

Lung stromal cells have also exhibited antigen presenting activity through mutual interaction with localized dendritic cells (63, 64), suggesting a role in long-term immune protection via sustaining effector resident memory T cell functionality. Notably, multiple additional studies have also reported that lung fibroblasts create a specialized niche that plays a key role in development and activation of lymphocytes following vaccination (65–67). Interestingly, a distinct phenotype of IL-33–producing fibroblasts was also identified when fibroblast stromal cells were differentially targeted by adenovirus vector–based vaccination, indicating a therapeutic potential for fibroblasts in vaccination against respiratory infections via supporting memory T cell inflation (68).

### Fibroblasts in acute lung infections.

In the context of viral infection, the ability of fibroblasts to activate virus-specific CD8^+^ T cells and to limit immune exhaustion following viral infection has been shown to depend on type I interferon signaling. This interferon-responsive regulation of CD8^+^ T cells by fibroblasts was observed in both lymph nodes and peripheral tissues, such as lung (51, 69, 70). In response to influenza virus infection, lung fibroblasts can adopt different activation states, including those associated with antiviral, proinflammatory, and repair functions. In cases of severe influenza infection, inflammatory lung fibroblasts may drive lethal immunopathology based on the activity of the ECM protease ADAMTS4, which is consistent in mouse models and severe human influenza infection (30). Signals associated with tissue damage, including IL-1 and TNF-α, can activate inflammatory fibroblasts during influenza infection. However, it is unclear whether these responses are preferentially activated in adventitial or alveolar fibroblasts and how compartmentalization of these responses would influence disease outcome.

Microbes and microbial metabolites were also shown to be directly sensed by fibroblasts through TLRs, resulting in modulation of inflammatory responses and subsequent activation of MyD88, an adaptor protein that triggers NF-κB signaling (71, 72). These fibroblasts were transcriptionally similar to those identified in mouse models of pulmonary fibrosis and described as pathologic. They also expressed the collagen triple helix repeat containing 1 (*CTHRC1*/*Cthrc1*) gene (13). Lineage tracing tools and studies in mouse models of pulmonary fibrosis identified alveolar fibroblasts as the source of *Cthrc1*-expressing pathologic fibroblasts (73).

Moreover, lung-derived macrophages from patients with COVID-19 exhibited a profibrotic transcriptional phenotype comparable to those identified in idiopathic pulmonary fibrosis (IPF), and exposure to SARS-CoV-2, but not influenza A virus, sufficiently induced a similar profibrotic phenotype in vitro, suggesting a unique fibroblast-gene signature in response to respiratory viral infection (74). Lung macrophages from patients with COVID-19 and those identified in IPF shared transcriptional signatures including expression of the profibrotic genes *SPP1*, *CD163*, and *MMP3*. Transcriptomic analyses predicted close interactions between lung fibroblasts and profibrotic macrophages, which were supported by immunostaining in patient lung tissue. The strength of these predicted fibroblast-macrophage interactions increased with days after symptoms began, consistent with fibroblast-macrophage activation being important for progression of postviral lung disease. Interestingly, angiotensin-converting enzyme 2 (ACE2), a receptor for SARS-CoV-2, has also been shown to be upregulated in lung fibroblasts from models of chronic, but not acute, lung inflammation, suggesting a direct contribution of lung fibroblasts to infection susceptibility in patients with COPD and IPF (75, 76).

In studies of lung tissue samples from human COVID-19 cases, activated fibroblasts were also associated with areas of pulmonary fibrosis in distal lung (77, 78). Notably, studies in human COVID-19 cases were performed on lung samples collected after development of severe disease and upon death of the subjects when failed tissue repair and fibrosis would most likely be present. For practical and ethical reasons, these studies were unable to capture fibroblast activation states early in infection and define the differentiation pathways that these cells adopt from initial resting state to a terminal pathologic state.

### Fibroblasts in acute respiratory distress syndrome.

Acute respiratory distress syndrome (ARDS) results from direct (as in infectious pneumonia) or indirect (as in extrapulmonary trauma) triggers and manifests clinically as the sudden onset of severe hypoxemia associated with pulmonary infiltrates in the absence of heart failure (79). Via production of cytokines, chemokines, growth factors, and ECM proteins, fibroblasts are essentially involved in coordinating host immune responses following exposure to lung insult (70, 80). Such contribution is driven by a transient transcriptional reprogramming in fibroblasts before returning to a resting state. The FGF/FGF receptor signaling pathway has been implicated in the pathophysiology of ARDS as well as additional respiratory disorders, including lung cancer, pulmonary hypertension, COPD, and fibrosis (81). The implication of growth factor signaling pathways in diverse pulmonary pathologies demonstrates their importance in regulating fibroblast responses to a variety of triggering signals in ARDS (82, 83). Several recent studies, however, suggest that acute triggers, driven by both infectious and sterile insults, activate fibroblasts and initiate differentiation to a pathologic, profibrotic state.

## Immunoregulatory functions of fibroblasts in chronic inflammation

**Figure 2:**
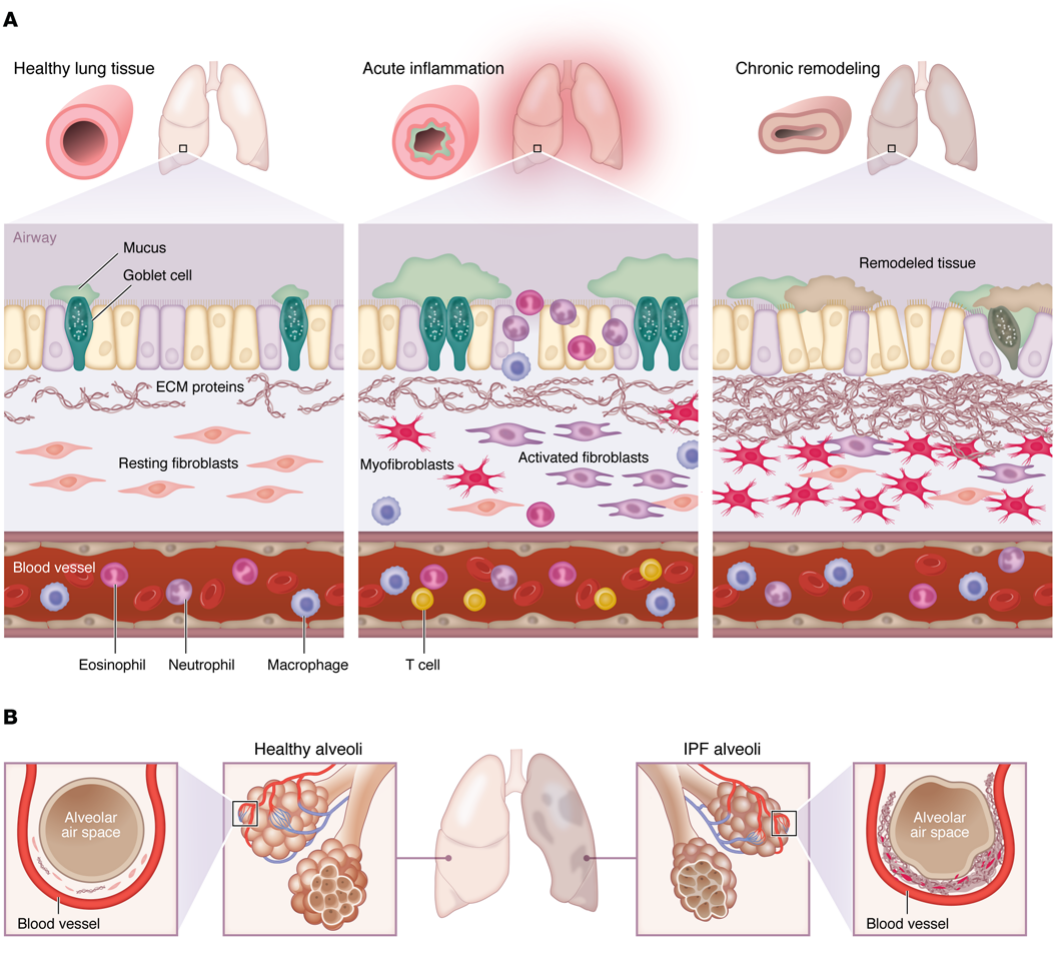
Fibroblasts exposed to chronic inflammation have a role in remodeling lung tissue. (**A**) Persistent stromal signaling facilitates the expansion of activated fibroblasts and their transition to myofibroblasts. ECM proteins are produced upon fibroblast activation. Together with soluble stimuli, deposited ECM proteins drive structural changes and promote multiple physical cues, including matrix stiffening that remodels lung tissue into phenotypes observed in ARDS, asthma, COPD, and IPF. (**B**) Alveolar fibrosis versus healthy alveoli.

Chronic lung inflammation is induced by persistent exposure to triggers followed by infiltration and retention of immune cells with a lack of repair signaling machinery. This process leads to an increased deposition of ECM proteins with excessive production of inflammatory mediators, including TNF-α, IL-6, and IL-8 in the respiratory tract (84, 85), consequently leading to structural alteration and remodeling as graphically demonstrated in Figure 2. While there are common mechanisms that fibroblasts utilize to modulate tissue immunity across chronic diseases, several subsets of fibroblasts are generally considered either location or disease specific. Our knowledge, however, regarding the specific role of these subsets has been limited by their intrinsic variability and the lack of selective markers (86). The vulnerability of the respiratory system to inflammation is usually attributed to an alteration in physiologic defensive mechanisms and the low regenerative capacity of the lungs, resulting in tissue injury, cell death, and respiratory dysfunction, all of which are main characteristics in asthma, COPD, and pulmonary fibrosis (87).

In models of pulmonary fibrosis, fibroblasts appear to transition through an inflammatory state, characterized by expression of immunoregulatory genes, as they differentiate into a fibrosis-promoting cell type (29, 73). The signals required to guide these differentiation processes remain unclear.

### Fibroblasts in asthma.

Bronchial asthma is among the most common chronic pulmonary disorders, affecting over 10% of the world’s population, and its prevalence continues to rise. The disease is characterized by a series of events, including increased epithelial and smooth muscle thickness, goblet cell hyperplasia, increased mucus secretion, abnormal deposition of ECM components in the basement membrane (BM), and angiogenesis, all of which lead to tissue remodeling (88). The inflammatory response is typically associated with bronchial hyperresponsiveness and airflow restriction (89). Fibroblast-to-myofibroblast transition is a hallmark of asthma, wherein myofibroblasts continue to reside within the tissue and actively engage in tissue remodeling by exerting contractile stress on ECM and surrounding cells. This response is further intensified by secreted growth factors, immunomodulatory cytokines and chemokines that play important roles in cell adhesion and leukocyte activation (88, 90). Asthma patients with detectable fibroblasts in bronchoalveolar lavage samples show a markedly thicker BM with an over 10-fold increase in CD34/CD45RO/α-SMA– and CD34/procollagen I–expressing cells, suggesting strong correlation between recruited fibrocytes and the BM thickness (91). The epithelium-derived IL-1α induces a proinflammatory response that alters fibroblasts’ ability to repair fibrillar collagen I via LOX, LOXL1, and LOXL2 activity, suggesting a direct contribution of fibroblasts in bronchial inflammation and tissue remodeling in asthma (92).

Moreover, the Th2-secreted cytokines IL-4 and IL-13 induce fibrosis by promoting fibroblast invasion in lungs of asthma patients. Such effects can be blocked by TGF-β1 and MMP inhibitors, suggesting a direct role in promoting invasive fibroblast phenotypes in people with asthma (93). In asthmatic bronchial fibroblasts, the effect of IL-13 has recently been linked to the pathologic involvement of the histone demethylase JMJD2B/KDM4B, which is a major contributor in the development of steroid-resistant asthma (94). Importantly, drugs routinely used to target inflammatory pathways in asthma have shown poor efficacy or no efficacy in inhibiting remodeling (95, 96).

### Fibroblasts in chronic obstructive pulmonary disease.

COPD is a leading cause of death worldwide and a major health burden given the lack of efficient therapeutics (97). Loss of elastic fibers from bronchial and alveolar walls is a major defining feature of COPD. Models of non-infectious lung inflammation have provided additional insight into the diverse activation states that fibroblasts can acquire and underlying molecular mechanisms regulating their activity. Multiple GWAS identified the gene encoding Hedgehog-interacting protein (*HHIP*) as a risk factor for development of COPD (98). Haploinsufficiency of *Hhip* resulted in activation of inflammatory pathways that potentiate IFN-γ production from CD8^+^ T cells with emphysema development and has been used to model COPD in mice with age-related lymphocytic inflammation and emphysema (99).

The fibroblasts isolated from patients with COPD exhibited secretory phenotypes characterized by upregulation of several inflammatory markers, in addition to the matrix proteoglycans versican and elastin (100). Consistent with models of inflammatory fibroblasts in proximal regions of the lungs that drive lymphocytic inflammation and emphysema, a recent study also reported that conditional deletion of *Hhip* in Gli1^+^ fibroblasts resulted in expansion of tissue-resident T cells and epithelial cell loss (101).

As a primary source of ECM deposition during chronic lung inflammation, fibroblasts coordinate fibrosis formation by interacting with various subsets of myeloid populations, thus triggering differentiation of mesenchymal stromal cells into myofibroblasts and activating the TGF-β signaling pathway (102). The dysregulation of TGF-β signaling is a major driver of remodeling in patients with COPD, suggesting an association with disease severity (103). While localization is essential for enabling interaction between fibroblasts and adjacent immune subsets, the excessive production of VEGF by lung-derived fibroblasts likely results in continuous remodeling that is observed in the distal lung compartments of patients with COPD (104).

In addition to TGF-β signaling, multiple immune subset–derived cytokines also contribute to disease progression. These factors include IL-17 and IL-22, both of which have been shown to stabilize TGF-β receptors on fibroblasts, thus enhancing TGF-β signaling and increasing sensitivity to TGF-β (105, 106). While IL-13 induces TGF-β signaling in macrophages, its role in fibrosis acts independently through excessive stimulation of stromal and parenchymal cells. The targeting of IL-13, IL-4R, and IL-13Ra1 has been implicated in a reduction of chronic inflammation in different settings of tissue injury, suggesting a critical role for the interaction between fibroblasts and type 2 cytokine signaling in the development of chronic airway inflammation and lung fibrosis (107–110).

### Fibroblasts in idiopathic pulmonary fibrosis.

IPF is a chronic form of fibrotic interstitial pneumonia characterized by excessive collagen deposition. There is a lack of understanding of underlying causes and pathways that contribute to IPF progression, yet cellular senescence has been linked to its pathogenesis (111). The senescence-associated secretory phenotype (SASP) proteins are produced by lung fibroblasts. Interestingly, several of these proteins are predominantly produced by fibroblasts from patients with COPD and IPF and have been directly implicated in a multitude of chronic inflammatory responses, suggesting a correlation between fibroblast phenotype and disease severity (112). Although spatial proximity is critical for allowing crosstalk between fibroblasts and immune subsets, some reports have shown that fibroblasts can efficiently produce regions in the matrix, termed “deformation fields,” that generate a dynamic force within the stromal layer, allowing signal transmission to distant immune subsets and facilitating migration to the affected sites (113, 114).

Unlike the neutrophil and M1 macrophage phenotype that defines the IL-1/IL-17A/TGF-β axis, the fibrotic pulmonary response is linked to a predominant infiltration of eosinophils and M2-like macrophages (115), and an abundance of Th2 cytokines in IPF lungs. This immune response has been linked to an imbalance between anti- and profibrotic mediators in the lungs (116, 117). Moreover, IL-25 and IL-33 could serve as key initiators of type 2–dependent fibrosis by triggering IL-4 and IL-13 production in innate lymphoid cells, T cells, eosinophils, and type 2–associated leukocytes (118–120). Other reports have also identified various immune subsets as critical contributors to the development of IPF (121–124). Interestingly, a phase II randomized study targeting IL-13 was not successful in patients with IPF (125), suggesting that Th2-mediated immunity might not be the sole contributor in the development of fibrotic responses in patients with IPF.

Taken together, these studies indicate that diverse respiratory exposures can activate convergent inflammatory pathways in fibroblasts that drive acute and chronic lung disease and identify mechanisms underlying exacerbations in several pulmonary disorders.

## Therapeutic potential of targeting lung fibroblasts

**Figure 3:**
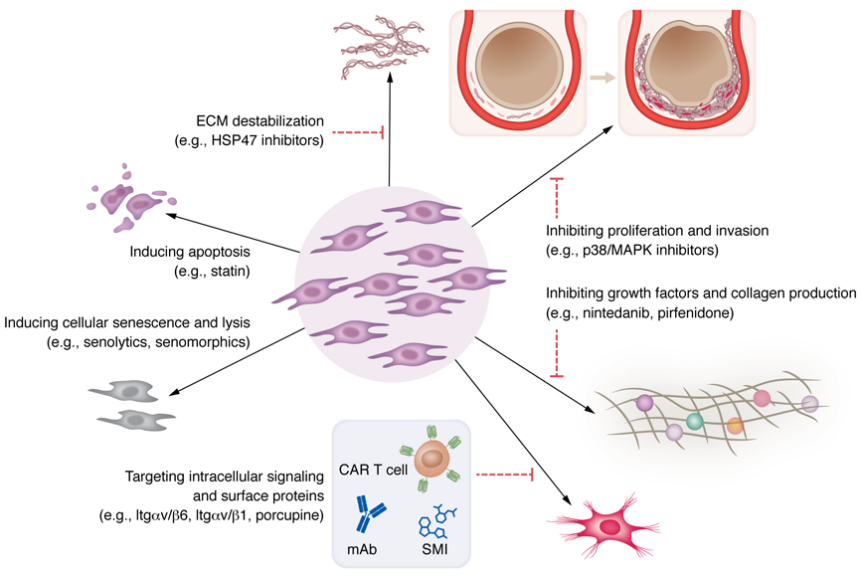
Therapeutic strategies that target fibroblast-mediated immune dysregulation may mitigate pulmonary inflammation and lung remodeling. Key therapeutic strategies for mitigating fibroblast-mediated immune dysregulation in pulmonary disorders include (a) promoting cellular senescence, (b) inhibiting proliferation and invasion, (c) inhibiting growth factors and collagen production, (d) inducing apoptosis, (e) destabilizing ECM, and (f) interfering with the fibroblast signaling machinery. SMI, small-molecule inhibitor.

Despite progress toward fundamental understanding of the pathogenesis of acute and chronic lung inflammation, many respiratory disorders still lack targeted disease-modifying therapies (80, 126–131). Multiple studies have recently demonstrated the potential of mesenchymal cells as a therapy in a range of disorders. Among mesenchymal cells, pulmonary fibroblasts and myofibroblasts are key targets (132–136). Our improved definition of fibroblast heterogeneity and response to inflammatory stimuli has expanded our understanding of their role in immune regulation. Indeed, a number of therapeutic approaches specifically directed at lung mesenchyme are currently being studied in a diverse array of respiratory disorders, including IPF, asthma, COPD, and ARDS. Figure 3 depicts key therapeutic mechanisms for mitigating fibroblast-mediated pulmonary disorders. In this section, we will highlight historical and contemporary therapeutic approaches and conclude with a view of the future, including potential strategies.

### Targeting fibroblasts in lung fibrosis.

A number of therapeutic approaches have been studied for mitigating COPD and IPF. Initially, and into the early 21st century, disease pathogenesis models focused on inflammation as the driver of dysregulated fibrotic responses (137, 138). Early reports suggested that subsets of patients achieved stabilization or even remission with corticosteroids (139, 140), which became the backbone of treatment strategies (141). Cyclophosphamide, a cytotoxic agent, or azathioprine, an antimetabolite agent, were subsequently added as treatment options (138). However, these treatments had little clear evidence of impact on inflammatory or fibrotic components of the disease (138). Eventually, the 2014 PANTHER study, a randomized double-blind trial of azathioprine, prednisone, and *N*-acetylcysteine (NAC) for IPF (142), had to be stopped early due to increased incidence of deaths, hospitalizations, and adverse events in the group receiving combination therapy (142).

Over the years, additional potential targets for therapeutic intervention have been trialed. NAC, a precursor to glutathione, was studied to reverse oxidative stress, a contributor to IPF pathogenesis. For a time, after the positive results of the 2004 IFGENIA study (143), which demonstrated improvements in lung function in patients treated with NAC in combination with prednisone and azathioprine, NAC was frequently prescribed by pulmonologists (144). However, the NAC therapy versus placebo arm of the study failed to demonstrate significant effects (145). Thus, NAC is no longer recommended as a monotherapy (146), but its potential benefits in subgroups of patients as an adjunctive therapy remain uncertain, and other therapies targeting oxidative stress are currently in development (147). IFN-γ-1b, which has preclinical data suggesting pleomorphic antifibrotic and immunoregulatory effects, was clinically tested with negative results (148). With these and other clinical trial experiences, IPF treatment focus shifted from antiinflammatory to antifibrotic responses.

Modulating fibroblast signaling has since become the contemporary mainstay of IPF treatment. In the recent past, antifibrotic medications, including colchicine, which also has antiinflammatory properties, and D-penicillamine, have been studied as potential treatment options (149). Following the 2011 CAPACITY trials (150) and the 2014 INPULSIS (151) and ASCEND (152) trials, nintedanib and pirfenidone have become the preferred treatment options for IPF. Each treatment targets a different fibroblast-profibrotic signaling pathway. Nintedanib is a small-molecule RTK inhibitor with activity against VEGFR, PDGFR, and FGF receptors, shown to slow lung fibrosis via reducing the production of collagen and profibrotic mediators (151, 153, 154). Pirfenidone is an antifibrotic agent that additionally mediates antiinflammatory effects via inhibiting TGF-β signaling and restraining fibrosis (152, 155). Despite the improvements offered by these agents, neither cures or reverses established fibrotic lung disease. Accordingly, a search for additional treatment regimens continues for this devastating disease.

There are a number of therapies in various stages of testing, from preclinical modeling to phase III clinical trials (156). Connective tissue growth factor (CTGF) inhibition via mAb blockade (157) and supplemental pentraxin-2 (158) have reached the phase III trial setting. Other candidate drugs in earlier phases of testing include inhibitors of integrin α_v_β_6_/α_v_β_1_ (146), porcupine (159), ectonucleotide pyrophosphatase/phosphodiesterase 2 (ENPP2), lysophosphatidic acid receptor (LPAR) (160), SRC kinase (161), and IL-11 (162). Finally, other approaches currently under evaluation include small interfering RNAs targeting hsp-47 (163), an aerosolized nucleic acid construct that inhibits TGF-β expression (164), targeting of the unfolded protein response (165), and cell senescence pathways (166), among others (167).

### Targeting fibroblasts in asthma.

Lung remodeling, most notably subepithelial fibrosis in the context of this Review, is a recognized feature of asthma. Current treatment recommendations are tailored toward reducing inflammation with inhaled corticosteroids and reversing bronchoconstriction with inhaled β-adrenergic receptor agonists or phosphodiesterase inhibitors. Though effective at reducing inflammation and bronchoconstriction, these commonly prescribed therapies do not prevent fibrosis or progressive tissue remodeling, which highlights the need for additional therapeutic approaches that combat this crucial feature of asthma (168).

As more experience is gained with biologics added to the asthma treatment armamentarium in the recent past, these drugs have been increasingly noted to effect lung remodeling (169). Mepolizumab, which targets IL-5, has been shown to be effective for asthma treatment (170) and additionally improves subepithelial ECM deposition (171). Omalizumab, an mAb targeting IgE receptor, may additionally act to reduce lung fibrosis (172). Though it has not been shown to demonstrate significant improvement in lung function in clinical trial settings (173), fevipiprant, which antagonizes the prostaglandin D_2_ receptor, decreases myofibroblast recruitment and eosinophilia, thereby reducing bronchial smooth muscle mass (174).

Other therapeutic targets of current interest and within the scope of this Review that additionally serve a dual role of reducing acute exacerbation and stabilizing or reducing lung remodeling include an MMP12 inhibitor (175), antibody-mediated IL-33 inhibition (176), and antibody-mediated thymic stromal lymphopoietin (TSLP) inhibition (177). Tezepelumab, an anti-TSLP mAb, was recently approved for asthma treatment after positive clinical trial results (177, 178). Though classically considered an alarmin product of bronchial epithelial cells, TSLP is additionally expressed by fibroblasts and is thought to play a role in fibrotic responses (179). Thus, there may be benefits of TSLP blockade that extend to amelioration of lung remodeling and fibrosis (180).

### Mesenchymal treatments in ARDS.

ARDS is thought to progress through a series of three stages: the exudative, the fibroproliferative, and, in a subset of ARDS survivors, the fibrotic phase (79). An exuberant or pathogenic fibroproliferative response, to which fibroblasts are key contributors, is thought to predispose to lung fibrosis. As such, fibroblasts, and their role in regulating reparative responses to ARDS, represent a relatively understudied potential therapeutic target for ARDS. It is notable that, even after years of clinical study, no currently approved disease-modifying drugs are available for ARDS, and treatment largely remains supportive in nature.

Corticosteroids of varying dosing regimens perhaps have the most published data regarding their effect as a disease-modifying agent for ARDS (181). Their use may be beneficial in certain disease phenotypes (182) as well as in patients who otherwise meet an indication for corticosteroids (183). However, it is likely that further studies incorporating contemporary lung-protective ventilation strategies along with corticosteroids will be required to determine their role in ARDS management. There is an ongoing randomized placebo-controlled trial studying pirfenidone to prevent post-ARDS pulmonary fibrosis as well as ongoing studies evaluating the roles of substrate-selective p38α inhibition (184), among others (highlighted in Table 2).

A final potential therapy to highlight for ARDS treatment within the scope of this Review is mesenchymal stem cells (MSCs) (185). MSCs may be obtained from one of several sources and notably have low immunogenicity, enabling allogeneic administration. MSCs may mediate pleiotropic beneficial effects on immune and structural lung cells in ARDS. Early phase I studies in patients with moderate to severe ARDS also demonstrate their safety (186); however, larger studies will be required to determine what role they may play in ARDS management.

## Future perspectives and therapies

The previous discussion summarizes some, but not all, of the potential therapeutic targets and pathways being explored for chronic inflammatory lung diseases in which fibroblasts play a key role in pathogenesis. The advent of single-cell transcriptomics has allowed for a more detailed understanding of basic fibroblast biology within the lung, in homeostasis and in the context of disease states. As these findings are further studied and validated, we anticipate that novel therapeutic targets will emerge. These data, coupled with a rapidly expanding armamentarium of biologics and cell-based therapies incorporated in treatments for other disease entities in which aberrant fibroproliferative responses play a role, such as in the immunosuppressive microenvironment of solid tumor malignancies (187), may enable incorporation of these treatment strategies to target deleterious fibroblast responses in inflammatory lung diseases.

## Author contributions

The order of the first authors is based on the significance of the contribution and on effort in writing the manuscript and in generating the supporting figures and tables.

## Figures and Tables

**Table 1:**
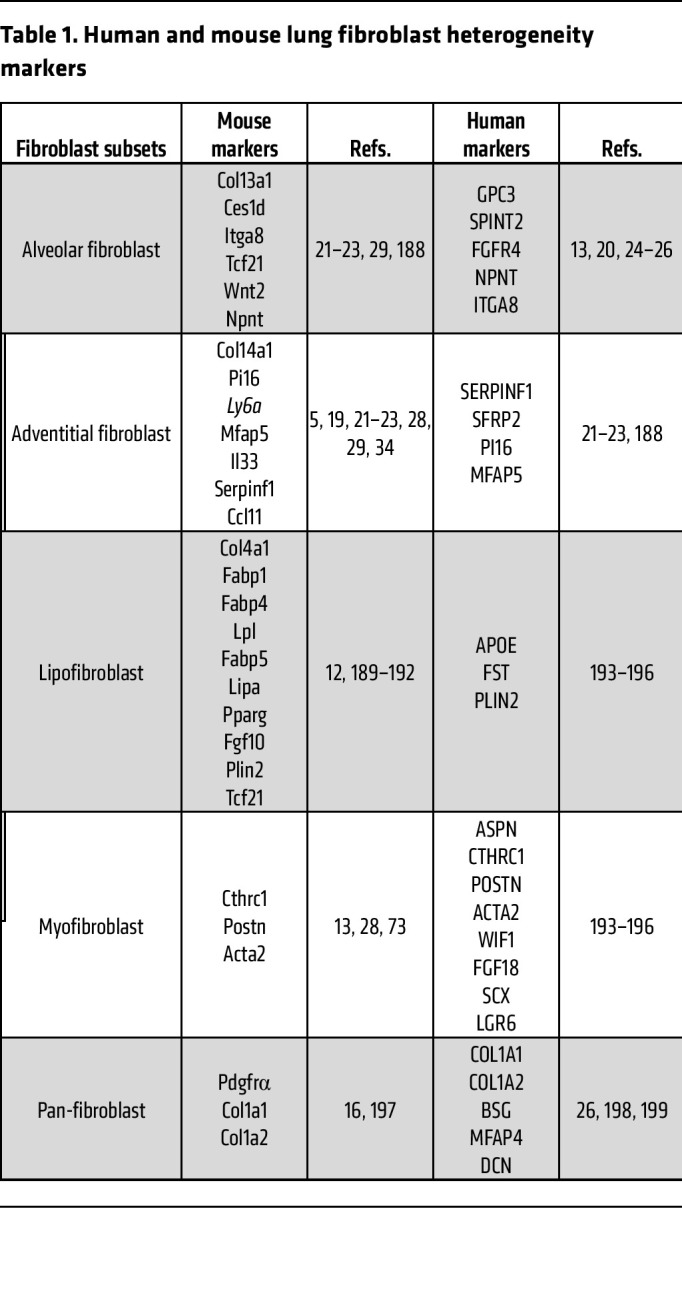
Human and mouse lung fibroblast heterogeneity markers

**Table 2:**
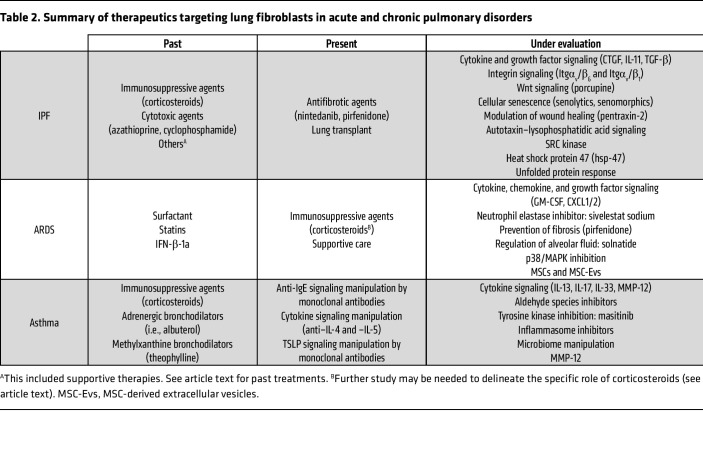
Summary of therapeutics targeting lung fibroblasts in acute and chronic pulmonary disorders

## Version 1 09/01/2023

Electronic publication
